# Image Guided Hypofractionated Radiotherapy by Helical Tomotherapy for Prostate Carcinoma: Toxicity and Impact on Nadir PSA

**DOI:** 10.1155/2014/541847

**Published:** 2014-03-18

**Authors:** Salvina Barra, Stefano Vagge, Michela Marcenaro, Gladys Blandino, Giorgia Timon, Giulia Vidano, Dario Agnese, Marco Gusinu, Francesca Cavagnetto, Renzo Corvò

**Affiliations:** ^1^Department of Radiation Oncology, IRCCS San Martino-IST, National Cancer Research Institute, 16100 Genoa, Italy; ^2^University of Genoa, DISSAL, 16100 Genoa, Italy; ^3^Department of Medical Physics, IRCCS San Martino-IST, National Cancer Research Institute, Genova, Italy

## Abstract

*Aim*. To evaluate the toxicity of a hypofractionated schedule for primary radiotherapy (RT) of prostate cancer as well as the value of the nadir PSA (nPSA) and time to nadir PSA (tnPSA) as surrogate efficacy of treatment. *Material and Methods*. Eighty patients underwent hypofractionated schedule by Helical Tomotherapy (HT). A dose of 70.2 Gy was administered in 27 daily fractions of 2.6 Gy. Acute and late toxicities were graded on the RTOG/EORTC scales. The nPSA and the tnPSA for patients treated with exclusive RT were compared to an equal cohort of 20 patients treated with conventional fractionation and standard conformal radiotherapy. *Results*. Most of patients (83%) did not develop acute gastrointestinal (GI) toxicity and 50% did not present genitourinary (GU) toxicity. After a median follow-up of 36 months only grade 1 of GU and GI was reported in 6 and 3 patients as late toxicity. Average tnPSA was 30 months. The median value of nPSA after exclusive RT with HT was 0.28 ng/mL and was significantly lower than the median nPSA (0.67 ng/mL) of the conventionally treated cohort (*P* = 0.02). *Conclusions*. Hypofractionated RT schedule with HT for prostate cancer treatment reports very low toxicity and reaches a low level of nPSA that might correlate with good outcomes.

## 1. Introduction

Many publications suggested that the *α*/*β* ratio (recognized as the ratio of “intrinsic radiosensitivity” to the “repair capability”) of prostate adenocarcinoma was comparable to that of late-responding normal tissues or even lower. If the estimated value for prostate *α*/*β* (1.5 Gy) [1] is reliably less than that for late-responding rectal damage (3 Gy), hypofractionation in the treatment of prostate cancer can offer an improved therapeutic ratio, due to a presumed higher sensitivity of prostate cancer tissues to higher fraction dose compared to the sensitivity of normal tissues damage. Randomized and prospective trials of hypofractionation treatment schedule for prostate cancer have confirmed excellent biochemical control rates and low toxicities [2–8]. These clinical studies used external beam hypofractionated regimens with dose-per-fraction ranging from 2.5 to 3.1 Gy delivered daily for 4–6 weeks. Concern remains over the use of such schedules with conformal radiotherapy technique (3D-CRT) because of the potential acute toxicity to normal organs close to the prostate. Today, with new technologies such as the use of image guided radiotherapy (IGRT) and dynamic intensity modulated radiotherapy (IMRT), it is possible to irradiate the target more accurately [9, 10] by reducing the volume of normal tissue irradiated, compared with conventional conformal (3D-CRT) techniques, while allowing to deliver higher doses to the clinical target. We present the results of a retrospective analysis of prostate cancer patients treated with hypofractionated IMRT by Helical Tomotherapy (HT) (Accuray, Inc, Sunnyvale, CA, USA). This hypofractionated schedule is radiobiologically isoeffective to 82 Gy with a conventional 2 Gy per fraction treatment (considering a prostate cancer *α*/*β* ratio of 1.5). The aims of our analysis were to assess acute and late toxicities of a hypofractionation regimen delivered with HT and investigate the radiobiological effects of mild hypofractionation on the value of PSA nadir (nPSA) and the time to reach nPSA nadir (tnPSA).

## 2. Materials and Methods

### 2.1. Patient Eligibility

In our Department, 120 men were treated with HT for prostate cancer between 2009 and 2013. All patients had a histological confirmed diagnosis of prostate cancer. Radical radiotherapy was planned for 80 (66%) patients; 17 (14%) patients underwent postoperative adjuvant radiotherapy and 23 (19%) were treated as patients that need salvage radiotherapy after biochemical relapse and/or clinical symptoms. For this report we analyzed only 80 patients treated with radical “up-front” radiotherapy. All patients were staged with ultrasound-guided biopsy; median prostatic sampling number was 10 (range, 6–18 samples). Patients with intermediate and high-risk disease also underwent bone scan and MRI (magnetic resonance imaging); in the postoperative and salvage radiotherapy the most recent cases received an additional staging with choline PET-CT (positron emission tomography-computed tomography). The median age was 72 years old (range: 53–82) with an ECOG-performance status value of 0-1 [16]. As for their risk-category (D'Amico) [17], 14 (17.5%) patients were at low risk (PSA ≤10 ng/mL; Gleason Score ≤6, and tumor category T1c-T2a), 28 (35%) were at intermediate risk (PSA > 10–20 ng/mL or Gleason Score = 7 or T2c), and 38 (47.5%) were at high risk (PSA >20 ng/mL or Gleason Score > 7 or two median risk factors). Androgen deprivation therapy (ADT) was administered to 54 (67.5%) patients before and after radiotherapy, for a mean duration of 22.5 months, range: 3–43. To evaluate the radiobiological effect of hypofraction on PSA kinetics, the nPSA and tnPSA of 26 patients treated with hypofractionation without ADT were retrospectively compared with those of a similar cohort of 20 prostate cancer patients treated by LINAC (linear accelerator) based conventional 3D-CRT with a standard dose of 76 Gy in 38 fractions. The clinical characteristics and initial PSA levels of all these patient populations are summarized in Tables 1 and 2. The two cohorts of patients, evaluated in this retrospective study, were treated during the same time lag between 2009 and 2013. The criterion of choice between hypofractionated and conventional fractionated schedule was due to the order of priority of patients' admission to our department and the availability of HT.

### 2.2. Treatment

Hypofractionated radiotherapy was delivered with HT. Setup and CT scan simulation were performed with the patient in supine position, placed in an appropriate fixation device (Combifix, Civco Medical Solutions, USA) using a 2.5 mm slice thickness, and covered the abdominal-pelvic area. Prior to CT simulation and each day before the treatment, patients followed instructions with a brochure on proper bowel and rectal preparation with partially full bladder and an empty rectum. CT data sets were sent for contouring on the Eclipse treatment planning system (Varian Medical System, Palo Alto, USA) and then exported using DICOM-RT (digital imaging and communication in medicine) format to Tomotherapy Planning System. For all patients Clinical Target Volume (CTV) consisted of the prostate and the seminal vesicles. The entire prostate was outlined as CTVp, seminal vesicles as CTVsv. Pelvic lymph nodes were outlined as CTVln following RTOG consensus guidelines [18] in all the patients at risk according to the Roach formula. To obtain the planning target volume of the prostate (PTVp) and the seminal vesicles (PTVsv), CTV was expanded isotropically with a 7 mm margin, except posteriorly where only a 3 mm margin was added. Rectum, bladder, femoral heads, large and small bowel, and penile bulb were outlined as organs at risk. The course of radiotherapy consisted of 27 fractions of 2.6 Gy daily for a total dose of 70.2 Gy. The dose was calculated with the formula NTD (normalized total dose) with a prostate *α*/*β* of 1.5 Gy. The volume of the seminal vesicles received a total dose of 60.75 Gy in 27 fractions, 2.25 Gy daily. If pelvic nodes were irradiated with adjuvant intent, a total dose of 50 Gy in 27 fractions with a single dose of 1.85 Gy per fraction was delivered. Dose was planned to cover the 95% of the PTV with at least the 95% of the prescription dose. Dose-volume histogram (DVH) goals for the rectum were such that the *V*40 ≤ 43%, *V*50 ≤ 32%, and *V*65 ≤10% were obtained (*Vx*: the percentage of target volume that received the *x* dose). The bladder DVH goals were *V*40 ≤47%, *V*55 ≤27%, and *V*60 ≤ 14%. The femoral head DVH goal was *V*20 <50%; a constraint to the bowel placed out of the PTV was accepted with a mean dose of 19.8 Gy. Megavoltage computed tomography (MVCT) by HT was performed every day before treatment to correct patient setup according to bone and soft tissue anatomy and to take into account intrafraction variability (e.g., due to over distension of the rectum). Treatment times were typically of 15–20 min. If patients were found with unacceptable bladder or rectal filling; the treatment was deferred to obtain correct filling volumes. For the cohort of 20 patients treated with 3D-CRT by a linear accelerator (Clinac 2100 CD, Varian, Palo Alto, CA, USA) the treatment was delivered with 38 fractions of 2.0 Gy to a total dose of 76 Gy. CTV were the same as those of hypofractionated cohort. CTV expansion to PTV that was 13 mm isotropically with exception of posterior expansions that were only 8 mm was added. PTV coverage goals were the same as previously mentioned for the patients treated with HT. Dose constraints to the rectum were *V*70 <20%, *V*60 <40%, and *V*50 <55%. Bladder constraints were *V*75 <25%, *V*70 <35%, and *V*65 <50%. Clinical setup was assessed with weekly electronic portal imaging device (EPID).

### 2.3. Follow-Up, PSA Nadir Evaluation, Toxicity Scoring, and Statistical Analysis

Patients were assessed weekly during treatment, 4 weeks after the end of treatment, and then subsequently every three months. The follow-up was performed with medical examination, PSA assay, and filling out a form for the detection of toxicity; imaging studies were prescribed only in those cases with abnormalities at diagnosis. Acute and late toxicity were assessed using the RTOG/EORTC (Radiation Therapy Oncology Group/European Organization for Research and Treatment of Cancer) radiation morbidity scoring criteria [18]; toxicity was defined to be acute or late if occurred within 3 months or after 3 months following the treatment, respectively. Adverse gastrointestinal (GI) and genitourinary (Gu) reactions were analyzed by incidence. The nPSA is defined as the lowest PSA value following radiotherapy. Biochemical failure (BF) was assessed using the nadir + 2 (Phoenix) definition [19, 20]. Statistical analyses were performed using JMP v 10.0 (SAS Institute, Cary, NC, USA). Cumulative incidence of biochemical failure (bF) and biochemical disease free survival (bDFS) was estimated by Kaplan-Meier method. An analysis between median nPSA of exclusive hypofractionated RT and exclusive 3D-CRT patients was carried out with Mann Whitney test for nonparametric data. Comparison of bDFS distribution between patients treated with or without ADT was calculated with Log-Rank Test.

## 3. Results

**Figure 1:**
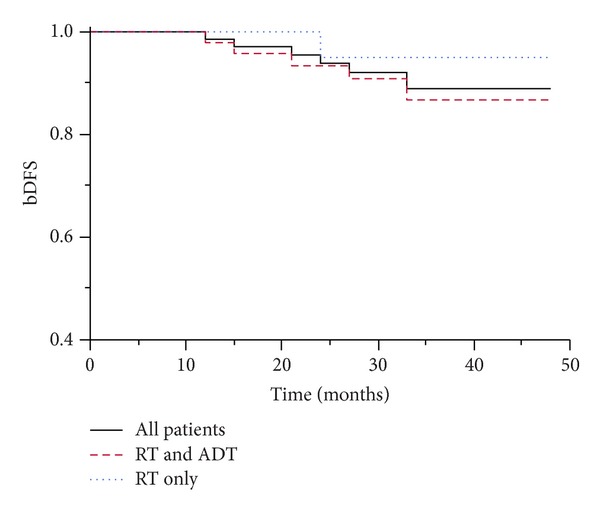
Biochemical disease-free survival (bDFS) in exclusive RT and ADT + RT.

**Figure 2:**
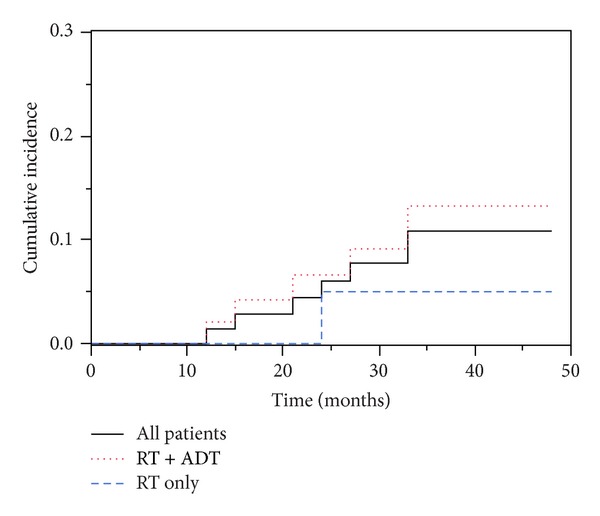
Incidence of biochemical disease failure (bDF) for exclusive RT and ADT + RT.

Data of eighty patients submitted to radical radiotherapy were eligible to be retrospectively analyzed for study. All patients completed the full treatment without any delays. No patients were lost for follow-up. The treatment plans provided excellent PTV coverage with an average of 98.4% of the PTV receiving 95% of the prescribed dose. With a median follow-up of 36 months (range 5–52) 79 patients were alive. A patient died from cancer unrelated cause at 24 months after radiotherapy. The 36-month bDFS was 88.9%. There was no significant difference in bDFS among patients treated with radiotherapy plus ADT and those treated with exclusive radiotherapy (95% versus 86.7%; *P* = 0.9). The cumulative incidence of biochemical failure at 36 months was 11% for the whole group of patients and, respectively, 5% in the RT group and 13.3 in the RT + ADT group (Figures 1 and 2).

### 3.1. Acute Toxicity

**Figure 3:**
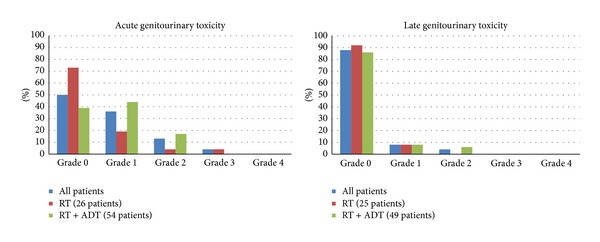
Genitourinary toxicity.

**Figure 4:**
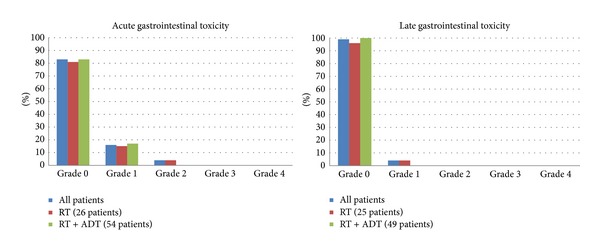
Gastrointestinal toxicity.

All patients were evaluable for acute toxicity. The treatment was well tolerated with 50% of patients with no GI toxicity and 83% no GU toxicity with no patient experiencing any grade 4 urinary or bowel toxicity; only one patient had a grade 3 GI toxicity. The results are reported in Tables 3 and 4 (or Figures 3 and 4). The most frequent symptoms during or soon after radiotherapy were urinary urgency, moderate increase of frequency, nicturia, and dysuria.

### 3.2. Late Toxicity

Median follow-up was 3 years (range 5–52 months); 74 (92.5%) patients were evaluable for late toxicity. 24/74 patients had been treated with radiotherapy alone and 50/74 with ADT also. No GU effects were reported in 65 (88%) patients (score 0) for RTOG-EORTC and 6 (8%) suffered minor effects (score 1). No GI problem was reported in 73 (99%) patients. A modest degree of toxicity (score 1) occurred in one patient. In Tables 1 and 2 we summarize the rates of urinary and rectal toxicity (incidence) observed at follow-up. As shown, there were no grades 3 and 4 toxicities. Grades 2 and 1 bladder toxicities were seen in 3% (4 patients) and 8% (6 patients), respectively. The only common factor between both patients with grade 2 genitourinary toxicity was frequency. None of the patients had urologic instrumentation procedures. Urinary incontinence, complete obstruction, or persistent hematuria was not observed. No patients had grade 3 GI toxicity; only one patient developed a grade 1. Persistent rectal bleeding was not observed. Toxicity rates compared between patients with or without hormone therapy showed no differences between the two groups. Erectile dysfunction evaluation was reported only in 63 cases. Erectile dysfunction before treatment was registered only by structured interview in 12 patients. During the follow-up 45 patients referred impotence, 30 were submitted to RT + ADT, and 15 to exclusive RT.

### 3.3. PSA Nadir and Time to PSA Nadir

**Figure 5:**
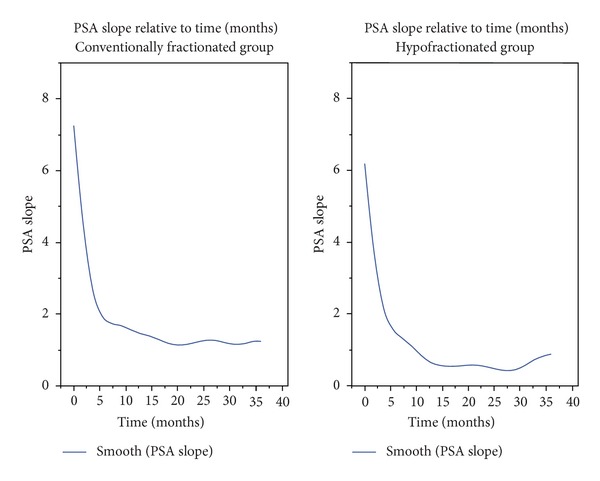
Trend of the PSA to achieve nadir.

**Figure 6:**
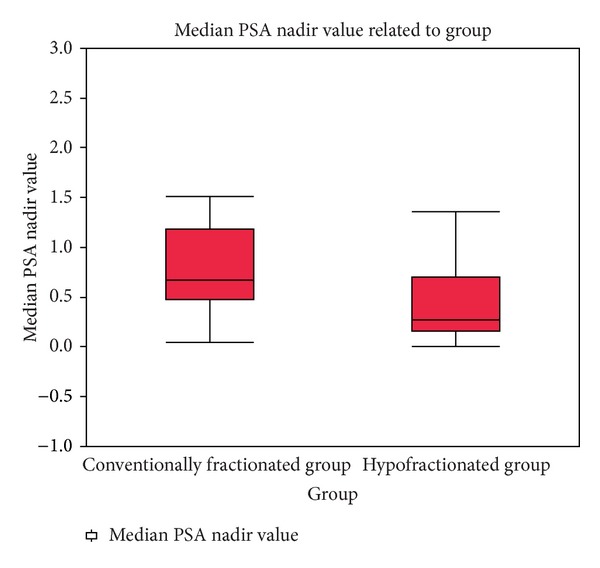
Boxplot of the median nadir PSA between patients treated with exclusive radiotherapy with a conventional fractionation and hypofractionation. The difference between the two groups of median PSA nadir is significative.

The patterns of PSA response after completion of radiotherapy showed a gradual decline. For the entire group (±ADT) of patients the average nPSA was 0.37 ng/mL, median 0.08 (SD 0.8) (standard deviation). We examined separately the values of the nPSA in the group of patients after exclusive RT and after RT plus ADT. The RT group average nPSA to date was 0.32 ng/mL and median 0.28 ng/mL (SD 0.4—range 0.01–1.36). One of the objectives of the study was to evaluate the nPSA. For the cohort of 80 patients we found an average and median values of 30 months. For the exclusive RT group the mean and median time of the nPSA were 30 months. To study whether a radical radiotherapy treatment delivered in less than 6 weeks by a hypofractionation schedule impact on nPSA differently from a 2 Gy daily fractionation schedule we collected the nPSA value and the corresponding time to nadir between a group of 20 patients treated with standard fractionation. The reported results for this group were average nPSa 0.86 ng/mL, median nPSA 0.67 ng/mL (SD 0.7; range 0.05–3.44); average tnPSA 18 months, median 27 months. Comparing the tnPSA and the median nPSA between patients treated with exclusive radiotherapy with hypofractionation or conventional fractionation no significant differences were observed between the times to reach the nadir (30 versus 27 months, *P* = ns), while the values of the nPSA were significantly lower in the group treated with hypofractionated RT (*P* = 0, 02) (Figures 5 and 6).

## 4. Discussion

We report our experience of hypofractionation with HT for prostate cancer. The study aimed to report the registered toxicity of hypofractionation with volumetric IG-IMRT technique and investigate whether hypofractionated schedule could have an impact on the nadir PSA and the time required reaching it. Our study has several strengths including the numerous detection of PSA after radiotherapy (every 3 months) for all the cohort of patients analyzed. Moreover few data have been published yet about the slope of PSA and nPSA after hypofractionated radiotherapy compared with conventional one. On the other hand we are well aware of the limitation of our study as the low number of patients evaluated and the short follow-up time. The HT is a unit of treatment with intensity-modulated beams equipped with a system of image guided integrated (IGRT). The megavoltage (MV) CT images acquired with the HT can be recorded and matched with the CT planning, immediately before irradiation, to correct errors in the setup and interfraction organ motion. The use of IGRT allowed the reduction of the expansion of CTV to 7 mm from 13 mm in all directions except posteriorly, where we expanded only 3–5 mm. The importance of IGRT in prostate cancer treatment has been recently evaluated in the report of Zelefsky et al. [21], where 186 patients have been treated with IGRT to a dose of 86.4 Gy. The target of the patients was corrected daily based on kilo-voltage imaging of implanted prostatic fiducial markers. This group of patients were retrospectively compared with a similar cohort of 190 patients treated in the same period with IMRT and with the same prescription dose without, however, implanted fiducial markers (non-IGRT). At median follow-up time of 2.8 years (range, 2–6 years) the authors found a significant reduction in late urinary toxicity for IGRT patients compared with the non-IGRT patients. The 3-year likelihood of grade 2 and higher urinary toxicity for the IGRT and non-IGRT cohorts were 10.4% and 20.0%, respectively (*P* < 0.02). The incidence of grade 2 and higher rectal toxicity was low for both treatment groups (1.0% and 1.6%, resp., *P* < 0.81). This study, also, reported an improvement in prostate-specific antigen relapse-free survival for high-risk patients treated with IGRT compared with non-IGRT (97% versus 77.7%; *P* = 0.05). Another study investigating the role of IGRT in 367 patients was reported by Singh et al. [22]: in this trial the irradiation was delivered by 3D-CRT, with and without IGRT. Compared with the non-IGRT group, improvement was noted in all dysfunctional rectal symptoms using IGRT. In multivariable analyses, IGRT improved rectal pain (*P* < 0.02), urgency (*P* < 0.01), diarrhea (*P* < 0.01), and change in bowel habits (*P* < 0.01). The toxicities reported from our study confirm that a hypofractionated course using HT for prostate cancer is associated with infrequent rates of clinically significant urinary and rectal toxicity. Our results are similar to the ones reported by Kupelian et al. [23] that using IMRT with daily transabdominal ultrasound image guidance and with a fractionation of 70 Gy/2.5 Gy for fraction reported gastrointestinal late toxicity of G2, G3, and G4 of 3.1%, 1.3%, and 0,1%, respectively, while the urinary toxicities of G2, G3 were 5.1% and 0.1%. Also Martin et al. [24] reported results of 92 patients irradiated with single dose of 3 Gy, total dose of 60 Gy, using IMRT and daily electronic portal image (EPID) with implanted fiducial markers. The gastrointestinal late toxicity of G2 was 4% and urinary toxicity of G2 was 3%. Lock et al. [25] report the study of 66 patients who received 63.2 Gy in 20 fractions over 4 weeks. Fiducial markers and daily ultrasound were used for image guidance. At 36 months acute grades 2 and 3 toxicities were 34% and 9% for GU versus 25% and 10% for GI symptoms. Late grades 2 and 3 toxicity for GU were 14% and 5%, and GI toxicity was 25% and 3%. However, despite these good toxicity results, recently Pollack et al. [7] reported data from a randomized clinical trial of comparison between hypofractionationated and conventional external beam RT for prostate cancer and suggest that patients with compromised urinary function before RT may not be candidate to hypofractionation due to the higher incidence of late GU toxicity. Many studies have also shown that greater PSA nadir (nPSA) levels are associated with an increased risk of biochemical failure, local failure, distant failure, progression-free survival, and disease-specific survival [26–30]. These studies have proposed various nPSA cutoffs ranging from 0.2 to 4.0 ng/mL as being predictive for outcomes in patients treated with external beam RT, brachytherapy, or the combination with and without androgen suppression. Some studies have also suggested that the time duration of the nPSA after RT is related to the outcome [31, 32]. Ray at al. [33] reported the result of 4839 patients with median follow-up of 6.3 years. All patients were treated definitively with RT alone to doses higher than 60 Gy, without ADT; nPSA was the lowest PSA measurement during the entire follow-up period. In this study the tnPSA was defined as the time from completion of RT to the nPSA date. Greater nPSA level and shorter tnPSA were associated with decreased biochemical or clinical disease-free survival (PSA-DFS) and distant metastasis-free survival (DMFS) in all patients and in all risk categories. The authors, also, correlated the total dose of irradiation with nPSA and tnPSA: the results suggest that a dose >70 Gy was associated with a lower nPSA level and longer TnPSA in all the patients. Johnson et al. have studied the nPSA and tnPSA for 410 patients after definitive high dose (>75 Gy) external beam radiotherapy without androgen deprivation. On univariate analysis both nPSA and tnPSA were predictive of freedom from biochemical failure, freedom from metastases, and prostate cancer specific survival (*P* < 0.0001). On Cox proportional hazards, a tnPSA <12 months did have worse prognosis as compared with longer tnPSA, but for those who achieved nPSA after 12 months the tnPSA was no longer prognostic [34]. In consideration of these crucial parameters able to predict the response to radiotherapy, we examined in our group of patients the value of the nPSA. Our findings achieved with hypofractionation appear to be interesting. The nPSA was very low (mean 0.37 ng/mL) in the whole group of patients treated with hypofractionation IG-IMRT. Twenty-five patients treated with RT alone were compared with a cohort of patients irradiated in the same period with conventional fractionation by 3-D CRT without IGRT. The median nPSA for patients with RT hypofractionated was 0.28 ng/mL after a mean time of 30 months versus 0.67 ng/mL after mean time of 27 months (*P* < 0.01) in the other group. This comparison, evaluated in a nonrandomized setting, remains of low scientific significance. However, the difference in the biological dose delivered in different treatment times (5.5 weeks versus 7.5 weeks) may have an impact on ultimate local control. Many literature data have evidenced the role of hypofractionation for the successful treatment of prostate cancer. Our results, although on a small series of patients treated with hypofractionated IMRT, are interesting since a very low nPSA was obtained after 30 months. Interestingly, with very short schedules where high doses per fraction (>7 Gy) are delivered for five fractions, the nPSA could be much more low. Recently some authors have published the first results after robotic radiotherapy with Cyberknife. In Table 5 we briefly report some preliminary results from the literature. With these new modalities the values of nPSA are very low being between 0.1 and 0.3 ng/mL while time to nadir has not still been reported. Waiting for the mature data of these experiences we have recently started a prospective clinical trial delivering 36.25 Gy in 5 fractions, 7.25 Gy per fraction over 10 days with HT for patients with low-risk prostate cancer. In this new trial the nPSA and tnPSA are the main surrogate end-points of radiation tumor control. However, only a well-designed randomized trial comparing mild and ultrashort hypofractionation could demonstrate or not a detectable difference in toxicity and ultimate cure between these new fractionation schedules.

## 5. Conclusions

The hypofractionation by IG-IMRT, reported in this analysis, was confirmed to be effective for the treatment of prostate cancer in all category risks. The use of IMRT and IGRT limits acute and late toxicity. Moreover, the value of nPSA obtained with our fractionation is very low. This evidence has an important predictive value on the long-term efficacy of radiotherapy. However a greater number of patients and with adequate follow-up are warranted to confirm these excellent findings; this mild hypofractionated schedule is now currently used at our Department for its safety and efficacy.

## Figures and Tables

**Table 1:** Patient characteristics.

	Hypofractionated group		Comparative 3D-CRT group
	RT 26 pts. (%)	RT + ADT 54 pts. (%)	RT 20 pts. (%)
Stage			
T1	7 (27)	14 (26)	13 (65)
T2	16 (62)	28 (52)	6 (30)
T3	3 (11)	12 (22)	1 (5)
N0	24 (92)	46 (85)	20 (100)
N1	2 (8)	8 (15)	0
Gleason score			
≤6	16 (62)	11 (20)	14 (70)
>6	10 (48)	43 (80)	6 (30)
Risk category			
Low	10 (38)	4 (7)	12 (60)
Intermediate	13 (50)	15 (28)	7 (35)
High	3 (12)	35 (65)	1 (5)

**Table 2:** Pretreatment PSA.

	Mean	Median	Range (ng/mL)
Hypofractionated group			
All patients	10.45	34.90	0.02–61.72
RT only	6.93	6.84	3.20–11.0
RT + ADT	12.10	7.50	0.02–61.72
Comparative 3D-CRT group			
RT only	7.74	7.39	3.80–12.77

**Table 3:** Acute and late genitourinary toxicity.

Toxicity	Grade 0	Grade 1	Grade 2	Grade 3	Grade 4
Acute (80 pts.)					
All patients	40 (50%)	29 (36%)	10 (13%)	1 (4%)	0
RT (26 pts.)	19 (73%)	5 (19%)	1 (4%)	1 (4%)	0
RT + ADT (54 pts.)	21 (39%)	24 (44%)	9 (17%)	0	0
Late (74 pts.)					
All patients	65 (88%)	6 (8%)	3 (4%)	0	0
RT (25 pts.)	23 (92%)	2 (8%)	0	0	0
RT + ADT (49 pts.)	42 (86%)	4 (8%)	3 (6%)	0	0

**Table 4:** Acute and late gastrointestinal toxicity.

Toxicity	Grade 0	Grade 1	Grade 2	Grade 3	Grade 4
Acute (80 pts.)					
All patients	66 (83%)	13 (16%)	1 (4%)	0	0
RT (26 pts.)	21 (81%)	4 (15%)	1 (4%)	0	0
RT + ADT (54 pts.)	45 (83%)	9 (17%)	0	0	0
Late (74 pts.)					
All patients	73 (99%)	1 (4%)	0	0	0
RT (25 pts.)	24 (96%)	1 (4%)	0	0	0
RT + ADT (49 pts.)	49 (100%)	0	0	0	0

**Table 5:** Median nadir PSA after stereotactic series with extreme hypofractionation radiotherapy for prostate cancer.

Author	Median FU (months)	Median nPSA (ng/mL)
Katz et al. [11]	51	0.1
Mcbride et al. [12]	44	0.2
Freeman and King [13]	60	0.3
King et al. [14]	32	0.5
Bolzicco et al. [15]	36	0.6 (exclusive RT)
This report*	36	0.18 (ADT + RT)
	36	0.08 (ADT + RT)
	36	0.28 (exclusive RT)

*Mild hypofractionation.
